# The Effect of pH on the Size of Silver Nanoparticles Obtained in the Reduction Reaction with Citric and Malic Acids

**DOI:** 10.3390/ma13235444

**Published:** 2020-11-29

**Authors:** Lukasz Marciniak, Martyna Nowak, Anna Trojanowska, Bartosz Tylkowski, Renata Jastrzab

**Affiliations:** 1 Faculty of Chemistry, Adam Mickiewicz University, 61-614 Poznan, Poland; lukasz.marciniak@amu.edu.pl (L.M.); martynan@amu.edu.pl (M.N.); 2 Centre Tecnològic de Catalunya, Chemical Technologies Unit, Eurecat, 43007 Tarragona, Spain; anna.trojanowska@eurecat.org (A.T.); bartosz.tylkowski@eurecat.org (B.T.)

**Keywords:** silver, citric acid, malic acid, nanoparticles

## Abstract

In colloidal methods, the morphology of nanoparticles (size and shape) as well as their stability can be controlled by changing the concentration of the substrate, stabilizer, adding inorganic salts, changing the reducer/substrate molar ratio, and changing the pH and reaction time. The synthesis of silver nanoparticles was carried out according to the modified Lee and Meisel method in a wide pH range (from 2.0 to 11.0) using citric acid and malic acid, without adding any additives or stabilizers. Keeping the same reaction conditions as the concentration of acid and silver ions, temperature, and heating time, it was possible to determine the relationship between the reaction pH, the type of acid, and the size of the silver nanoparticles formed. Obtained colloids were analyzed by UV-Vis spectroscopy and investigated by means of Transmission Electron Microscope (TEM). The study showed that the colloids reduced with citric acid and malic acid are stable over time for a minimum of seven weeks. We observed that reactions occurred for citric acid from pH 6.0 to 11.0 and for malic acid from pH 7.0 to 11.0. The average size of the quasi-spherical nanoparticles changed with pH due to the increase of reaction rate.

## 1. Introduction

The global market value of nanotechnology is expected to reach $90.5 billion by 2021 as commercial and consumer nano-products continue to rise [1]. According to the recently published statistics silver is considered to be one of the most investigated and commercialized nanomaterials due to its good conductivity, chemical stability as well as catalytic and antibacterial activity [2,3,4,5,6,7]. During the last decade, design and fabrication of silver-based next-generation nanomaterials have been subjects of intense research in the field material sciences [8,9]. 

Indeed, silver and silver-based nanomaterials, as the fruitful results of these studies, have been widely applied in disinfection of medical devices and household appliances, catalysis, cosmetics, in wound dressings, for water treatment, and in various textile materials containing silver nanoparticles in fibers [6,7,10,11,12,13,14,15]. Moreover, published data indicate the “advantageous position” of silver nanoparticles around other bactericidal agents, due to their relatively cheap production cost and high efficiency [10,13].

In recent years, increasingly attention has been paid to green chemistry in design and production of nanomaterials [6,16]. Therefore, the interest of many researchers has been focused on the use of natural compounds or extracts from plants and fruits, as novel, alternative and breakthrough reducers and stabilizers [15,16,17,18,19]. In fact, compounds such as: fruit acids, naturally occurring in fruit, wine, and almonds, meet the requirements of green chemistry [20]. They are also called as alpha-hydroxy acids due to the presence of hydroxyl groups at the α-carbon atom as well as a variable number of carboxylic groups. They have been widely employed in cosmetics and dermatology (i.e., in skin moisturizing) for reduction of wrinkles and chemical peeling of the skin [20,21].

According to the published articles, it can be estimate that approximately 10% of protocols for silver nanoparticles production have employed sodium salt of citric acid as a reducing agent [22]. For the first time, the synthesis of silver colloids with sodium citrate was carried out in 1982 by Lee and Meisel [16]. The authors developed a procedure according to which an appropriate amount of aqueous solution of sodium citrate should be added to a boiling aqueous solution of silver nitrate. Then, the solution should be heated for 1 h, and finally it should be cooled down to room temperature [23,24]. It is worth to underline that by following this protocol citrate ions serve as both a reducing agent and a stabilizer, and they can coordinate Ag^+^ ions or Ag^2+^ dimers in the early stages of the reaction [25,26]. This method is still very popular, due to its simplicity (i.e., it does not require a specialized, sophisticated equipment) and high efficiency in high amount of silver colloid preparation; however, the obtained nanoparticles have a wide range of sizes and shapes [24,25,26]. It has been disclosed that by controlling the pH value of the reaction system, it is possible to successfully reduce the disadvantages of the mentioned method and to achieve nanoparticles with a more monodisperse distribution [27,28]. According to our knowledge, based on the literature/current state of the art investigation, the synthesis of silver nanoparticles in aqueous solution at various pH with citric acid [28,29] and ascorbic acid [27] has not been deeply studied. Published results demonstrated that conducted experiments were carried out only in a narrow pH range or with the use of an additional stabilizing agent.

In this article, we present results of the synthesis of silver nanoparticles using citric acid and malic acid in water, following to the modified method of Lee and Meise, in a wide pH range from 2.0 to 11.0, without addition of additives or stabilizers.

## 2. Materials and Methods 

Silver nitrate (AgNO_3_, ≥99%), citric acid (C_6_H_8_O_7_, ≥99%), DL-malic acid (C_6_H_8_O_7_, ≥99%), sodium hydroxide (NaOH, 99%), nitric acid (HNO_3_, 65%) and hydrochloric acid (HCl, 35–38%) from Sigma Aldrich were analytical grade and used without further purification. Ultra-pure water (Milli-Q, resistivity at 25 °C, 18.2 MΩ·cm) was used in all experiments. The glassware used in the synthesis were cleaned by aqua regia (HCl:HNO_3_ in 3:1 volume ratio), rinsed with plenty of deionized water, and dried.

The spectra of the silver colloids were recorded at a 1:1 dilution with deionized water at 20 °C in a PLASTIBRAND PMMA cell with 1 cm path length with an Evolution 300 UV–Vis ThermoFisher Scientific spectrometer (Jeol Ltd., Tokyo, Japan) equipped with a xenon lamp (range 300–1000 nm, accuracy 1 nm, sweep rate 240 nm/min). 

Morphology of silver colloids was analyzed by JEOL JEM 1011 TEM Electron Microscope (Thermo Electron Scientific Instruments LLC, Madison, WI, USA), with an acceleration voltage of 100 kV. Silver colloid was put on the Formvar Carbon Film 200 square mesh copper grids and dried in air. The size of the silver nanoparticles was determined using the ImageJ software and size distributions were prepared in Origin software.

## 3. Results

The synthesis of silver colloids was carried out according to the modified method of Lee and Meisel and proceeded in two steps [23]. At first, the citric acid/malic acid solution was adjusted to a specific pH (from 2.0 to 11.0) using a Titrino 702 Methron system (Metrohm AG, Herisau, Switzerland) equipped with a glass pH electrode, by adding the appropriate amount of 0.1892 M sodium hydroxide or 0.1 M nitric acid. The low value of the protonation constants of α-hydroxy acids caused the deprotonation of carboxyl groups to occur at low pH values. As can be seen in the distribution curves of the forms calculated on the basis of protonation constants, citric acid was completely deprotonated and at pH around 7.0, Figure S12 and in the case of malic acid at pH 6.0, Figure S13 (Supplementary Materials) [30,31]. In the second step, 49.5 mL of citric acid/malic acid solution at the specified pH was heated in a round bottom flask equipped with a reflux condenser, placed in an oil bath on a magnetic stirrer. After reaching the boiling point, 0.5 mL of 0.05 M silver nitrate was added and heating was continued for 60 min. Then, the heating was turned off, the flask was removed from the oil bath. Next, the colloid solution was cooled down slowly at room temperature. In the reactions, the concentrations of silver nitrate and citric/malic acid were $5\times 10^{-4}$ M and $5\times 10^{-3}$ M, respectively. The metal salt to reducer ratio was 5:1. No additional stabilizers were added to the colloids.

**Scheme 1:**
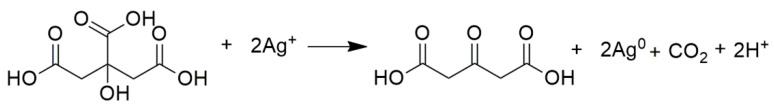
The citric acid was oxidized to acetone dicarboxylic acid.

**Scheme 2:**
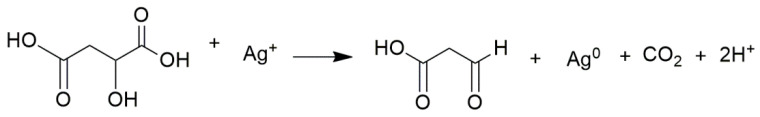
Malic acid was oxidized to 3-oxopropanoic.

The reaction for citric acid occurred from pH 6.0 to 11.0, while for malic acid it occurred from pH 7.0 to 11.0. Both acids in the reaction served as a reducing agent and stabilizer [24,26]. In the reaction, the citric acid was oxidized to acetone dicarboxylic acid (Scheme 1) [24], whereas malic acid was oxidized to 3-oxopropanoic acid (Scheme 2). 

### 3.1. Silver Nanoparticles Reduced by Citric Acid

**Figure 1:**
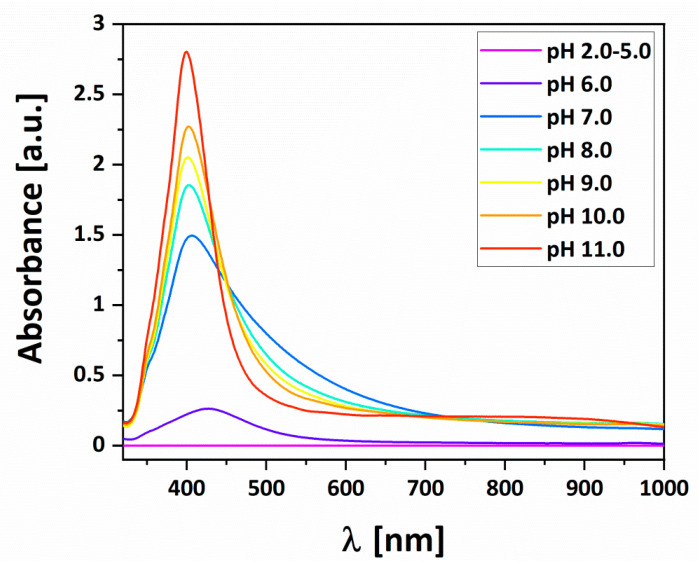
UV-Vis spectra of the silver nanoparticles reduced by citric acid at pH 2.0 to 11.0 in 1:1 dilution with deionized water.

Figure 1 presents UV-Vis absorption spectra of the silver colloids reduced by citric acid from pH 2.0 to 11.0, recorded 60 min after switching off the heating. Products synthesized at pH 6.0, 7.0, 8.0, 9.0, 10.0, and 11.0 demonstrated absorption peaks at 428, 407, 403, 401, 402, and 400 nm, corresponding to full width at half-maximum (fwhm) of 126, 145, 103, 90, 84 and 69 nm. 

Colloids were stored in dark glass bottles at room temperature and for 7 weeks tested their stability over time by weekly UV-Vis measurements (see Table 1). Before each measurement, the colloid solution was diluted to exclude the effect of dilution on stability and that could be a reason for the little fluctuations in the absorbance value. 

**Figure 2:**
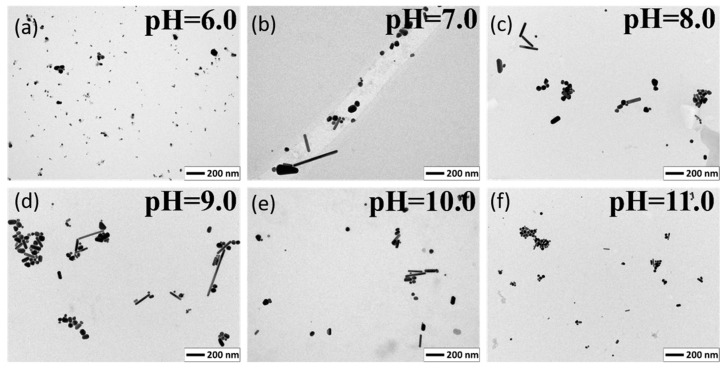
TEM images of the silver nanoparticles reduced by citric acid at pH 6.0 to 11.0. (**a**) pH = 6.0; (**b**) pH = 7.0; (**c**) pH = 8.0; (**d**) pH = 9.0; (**e**) pH = 10.0; (**f**) pH = 11.0.

Measurements using the Transmission Electron Microscope (TEM) were carried out to determine the size and shape of the obtained nanoparticles. The average sizes of the quasi-spherical nanoparticles were 11.81 ± 8.05, 43.18 ± 24.04, 35.49 ± 16.25, 36.04 ± 13.93, 36.24 ± 12.52, 25.33 ± 7.58 nm for pH 6.0, 7.0, 8.0, 9.0, 10.0, and 11.0 respectively. Figure 2. shows TEM images of the silver colloids reduced by citric acid, the scale bare is 200 nm. At pH 6, only small nanoparticles were visible, whereas from pH 7.0 to 11.0 the average size of the nanoparticles decreased with increasing pH. The largest particles were observed are at pH 7.0, while the smallest were observed at pH 11.0. 

**Figure 3:**
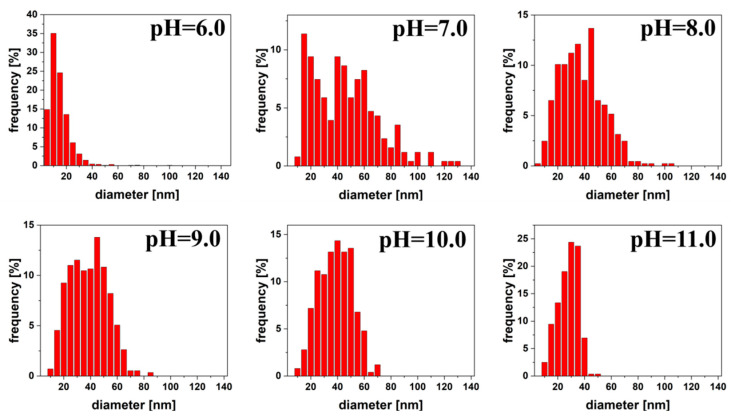
Size distribution of silver quasi-spherical nanoparticles reduced by citric acid at pH 6.0 to 11.0.

On the basis of a minimum of 200 measurements, size distributions were plotted. At pH 6.0, we observe a very narrow distribution of the size obtained nanoparticles, while at pH 7.0 it was very wide. From pH 7.0 to 11.0 we clearly see that the size distributions were getting narrower, and thus the polydispersity of the colloid decreased. Size distributions for the nanoparticles are shown in Figure 3. The same scale in nm on the *x*-axis was used for all size distributions for greater transparency.

**Figure 4:**
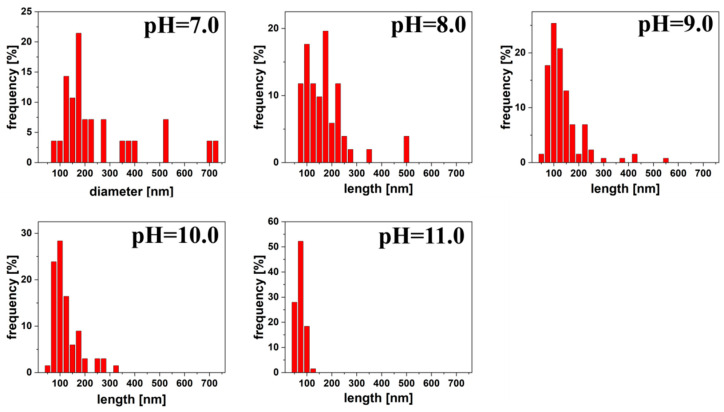
Distribution of rods length for silver colloids reduced by citric acid at pH 7.0 to 11.0.

In addition to the spherical shapes, in the TEM images, we can also see other shapes, mainly rods. They occurred from pH 7.0 to 11.0 and represented 9.89%, 10.26%, 18.49%, 20.32%, and 19.48% for pH 7.0, 8.0, 9.0, 10.0, and 11.0, respectively of all measured nanoparticles. As we can see in Figure 4. the distribution of rod length was gradually narrowed and size decreased as pH increased. The average size was 234.65 ± 172.98, 157.00 ± 88.26, 123.84 ± 75.10, 110.00 ± 56.63, 61.51 ± 15.40 nm for pH 7.0, 8.0, 9.0, 10.0 and 11.0 respectively. 

**Figure 5:**
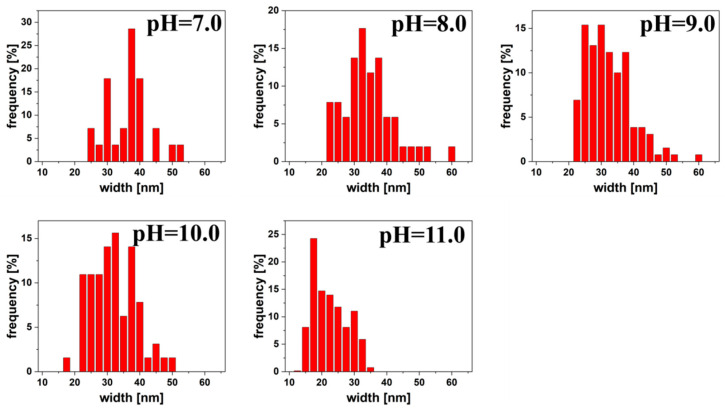
Distribution of rods width for silver colloids reduced by citric acid at pH 7.0 to 11.0.

However, for the width of the rods, there was no such clear difference as pH increases (Figure 5). The average width was 35.30 ± 6.90, 33.24 ± 8.01, 31.09 ± 7.05, 30.71 ± 6.85, 21.02 ± 5.35 for pH 7.0, 8.0, 9.0, 10.0, 11.0, respectively. The width of the rods was relatively constant from pH 7.0 to 10.0 and slightly decreased, and for pH 11.0 it dropped by more than 10 nm.

### 3.2. Silver Nanoparticles Reduced by Malic Acid

**Figure 6:**
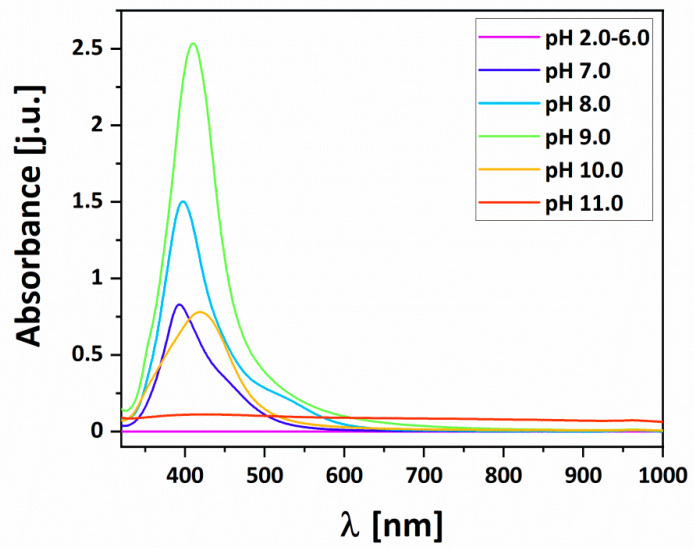
UV-Vis spectra of the silver nanoparticles reduced by malic acid at pH 2.0 to 11.0 in 1:1 dilution with deionized water.

Figure 6. shows UV-Vis absorption spectra of the silver colloids reduced by malic acid from pH 2.0 to 11.0, which were recorded 60 min after switching off the heating. The maxima of absorption peaks for pH 7.0, 8.0, 9.0, 10.0, and 11.0 were 393, 397, 409, 422, and 429 nm respectively, and corresponding to the full width at half-maximum (fwhm) of 71, 71, 69, and 85 nm; at pH 11.0 it was not possible to determine.

These colloids were also stored temperature in dark glass bottles in the dark at room temperature and for 7 weeks tested their stability over time by weekly UV-Vis measurements, using a new portion of the colloid (see Table 2). Before each measurement, the colloid solution was diluted to exclude the effect of dilution on stability. That could be a reason for the little fluctuations in the absorbance value.

**Figure 7:**
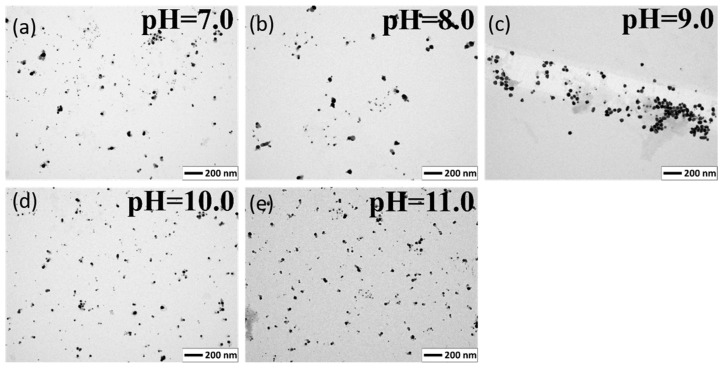
TEM images of the silver nanoparticles reduced by malic acid at pH 7.0 to 11.0. (**a**) pH = 7.0; (**b**) pH = 8.0; (**c**) pH = 9.0; (**d**) pH =10.0; (**e**) pH = 11.0.

To determine the size and shape of the obtained nanoparticles, a Transmission Electron Microscope was applied (Figure 7, scale bare is 200 nm). The average size of the quasi-spherical nanoparticles was 10.59 ± 7.94, 18.91 ± 13.65, 29.84 ± 13.07, 11.68 ± 8.76, and 13.97 ± 7.83 nm for pH 7.0, 8.0, 9.0, 10.0, 11.0 respectively. In all pHs except 9.0, we observed very small nanoparticles next to large ones, which lowered the average and increased the standard deviation. From pH 7.0 to 9.0, the average size of the nanoparticles increased and then dropped sharply, as did the absorbance of the colloids. At pH 11.0, it was difficult to draw conclusions because the colloid had a low absorbance due to the deposition of most of the reduced silver on the flask.

**Figure 8:**
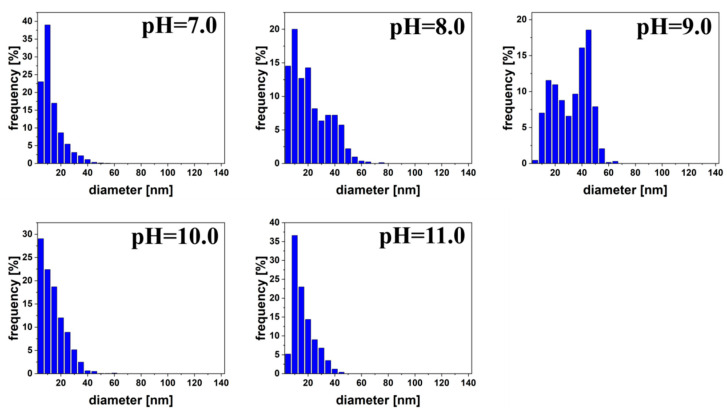
Size distribution of silver quasi-spherical nanoparticles reduced by malic acid at pH 7.0 to 11.0.

Size distributions of the nanoparticles are shown on Figure 8. For pH 7.0, 8.0, 10.0, and 11.0, the size distribution had a very similar shape and was shifted towards smaller values. However, at pH 9.0 we observed two maxima, the first at about 15 nm, and the second at 42.5 nm. The same scale in nm on the *x*-axis was used for all size distributions for greater transparency.

## 4. Discussion

**Figure 9:**
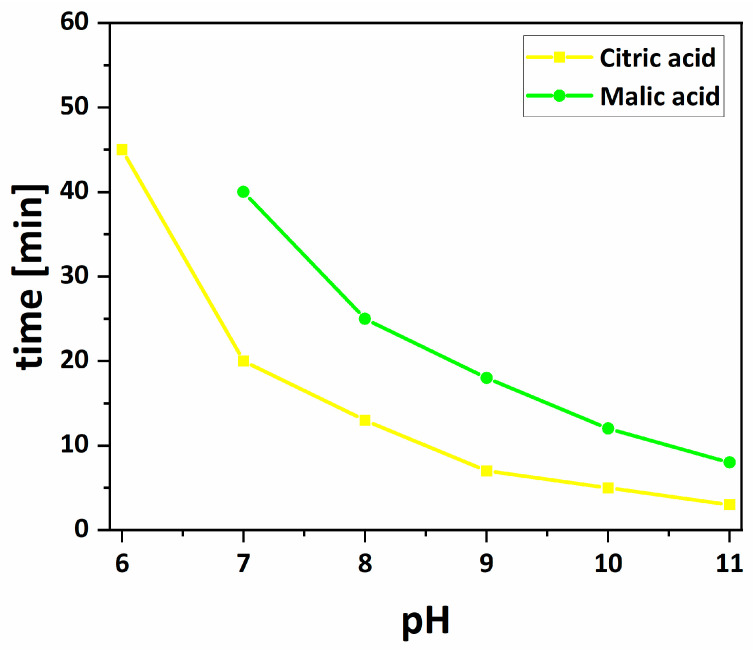
The dependence of time after which the reaction was observed as a function of pH for silver nanoparticles reduced by citric and malic acids.

Absorption peak of silver colloids reduced by citric acid were slightly shifted to shorter wavelengths and became much narrower with increasing pH, due to a decrease in the size and polydispersity of the nanoparticles [27,32,33]. Absorbance gradually increases with increasing pH. The biggest difference in absorbance is between pH 6.0 and 7.0, which is probably due to the fact that the reaction at pH 6.0 occurs after 45 min, while at pH 7.0 just after 20 min (Figure 9). The reason for this is that at pH 6.0, citric acid is a partially deprotonated form (pK_a1_ = 3.2, pK_a2_ = 4.8, pK_a3_ = 6.4) and cannot strongly coordinate silver ions and reduce them [24,34]. Therefore, the reduction reaction occurs after a longer time for pH 6.0 than for pH 7.0. For colloids reduced by malic acid, the absorption peaks are shifted to longer wavelengths from 7.0 to 11.0 and the absorbance increased from pH 7.0 to 9.0, and then at pH 10.0 dropped sharply, at pH 11 is a flat line, due to the increasing reaction rate and the weaker stabilizing properties of malic acid. Full width at half-maximum is more or less constant for pH 7.0–9.0, and it grows at pH 10.0. Malic acid has also weaker reducing properties, as indicated by the fact that the reaction at a given pH takes longer than for citric acid. With increasing pH, the reaction rate increases for both acids.

Stability of the colloids reduced by citric acid can be explained by the fact that citrate ions act also as stabilizers in the reduction reaction—stabilized nanoparticles by charge repulsion and are weakly bound to the silver producing a charged layer that serves as an electrostatic barrier to aggregation [7,34]. At pH 6.0, there is a slight increase in absorbance that could not be the result of the fluctuations associated with dilution. This is due to the fact that the solution contains unreduced silver ions and at room temperature citric acid is capable of reducing Ag^+^ ions, but it is a very slow process. That is why we can observe the increase of the absorbance at pH 6.0 after a few weeks [35]. From pH 7.0 to 11.0, there is a practically constant value of absorbance and only a small, up to several nanometers, fluctuation in λ_max_ at longer wavelengths, which is evidence of stability. Therefore, they do not contain unreduced silver ions, because there is no increase of the absorbance. 

The colloids obtained from pH 7.0 to 10.0 reduced by malic acid were found to be stable for at least 7 weeks. At pH 7.0, there is a little increase in absorbance and this is a similar situation as at pH 6 for nanoparticles reduced by citric acid. The solution still probably contains silver ions, which are reduced over time by malic acid. However, at pH 8.0 to 10.0, there is a practically constant value of absorbance during time and small, up to several nanometer fluctuation in λ_max_, which is evidence of stability. At pH 11.0, there was no sense to conduct stability tests due to the insignificant absorbance of the colloid. Malate ions act also as stabilizers in the reaction like citrate and stabilize nanoparticles by charge repulsion.

For silver colloid reduced by citric acid at pH 6.0, we observed small spherical nanoparticles, which was influenced by a short reaction time (Figure 9). From pH 7.0 to 11.0, the average size of the spherical nanoparticles decreased, but from pH from 8.0 to 10.0 the average size was nearly constant. The size of the silver nanoparticles reduced by malic acid changes with the pH of the reaction. It increased in the pH range from 7.0 to 9.0 and then decreased rapidly at pH 10.0 and 11.0. This is probably due to the fact that malic acid is a weaker reducer than citric acid, the reaction with it occurs later at the same pH, and the nanoparticles do not have time to grow. 

The obtained results for pH 7 to 11 for citric acid confirm the literature data [28,29]. With the increasing of pH, the reaction rate increases and particle size decreases. The explanation why at pH 6 we observe only small nanoparticles, and not greater than at pH 7, is the fact that all reactions were carried out for an hour after the addition of the solution of silver nitrate, while the literature does not specify the exact reaction time. However, for malic acid, there are no literature data on the reaction, where it would be both a reducing and stabilizing factor.

The presence of many rods in colloids reduced with citric acid at pH 7.0 to 11.0 is related to the fact that citrate ions bound more strongly to Ag (111) than Ag (100) surfaces and the nanoparticle can grow in the (110) direction to form a rod [24,36,37]. Theoretical calculations confirmed that citric acid may preferentially bind to the facets of Ag (111) due to the fact that the three-fold symmetry of citric acid is consistent with Ag (111) facets and results in four Ag-O bonds, whereas with Ag (100) facets citrate creates only two bonds due to geometry mismatch [37,38]. At pH 6.0, we did not observe the rods, probably because the nanoparticles are very small, and they just started growing and the rods would appear after a longer time of heating. In colloids reduced by malic acid, we do not observe rods because of the mismatch geometry of malic acid and silver.

## 5. Conclusions

As a result of reduction with citric, and malic acids according to the modified method of Lee and Meisel, we obtained quasi-spherical nanoparticles, in the case of citric acid rods. The reactions were carried out at pH 2.0 to 11.0; however, reactions occurred for citric from pH 6.0 to 11.0 and for malic acid from pH 7.0 to 11.0 respectively. The average size of the quasi-spherical nanoparticles changed with pH—for citric acid it decreased with increasing pH from 7.0 to 11.0, whereas for malic acid grew from pH 7.0 to 9.0, and then rapidly decreased. The time dependence after which the reaction was observed for both acids had a visible exponential relationship. Rods that appeared in colloids reduced by citric acid from pH 7.0 to 11.0, and their average size in length and width decreased with increasing pH. The study showed that the obtained colloids reduced with citric acid and malic acid are stable over time for a minimum of 7 weeks. Compared to the time after which the reaction was observed and that the reduction reaction occurs with malic acid at pH 7.0, we found that malic acid is a weaker reducer than citric acid. This is probably due to the weaker interaction between malic acid and Ag and due to the lower number of carboxylic acid groups in malic acid than in citric acid [19].

The obtained results allow to better see how the pH influences the formation of silver nanoparticles in the reaction with citric/malic acid. We demonstrate how the reaction rate is changing with pH and thus how the absorbance, size, and shape of nanoparticles changes. This will allow a better understanding of the influence of pH on this type of reaction with one compound, which is a reducer and stabilizer, and the design of a synthesis that will allow obtaining colloids of a specific size.

## Figures and Tables

**Table 1:** Comparison of data on the stability study of colloids reduced by citric acid determined using a UV-Vis spectrometer.

		Synthesis Day	1 Week	2 Weeks	3 Weeks	4 Weeks	5 Weeks	6 Weeks	7 Weeks
pH 6.0	λ_max_ (nm)	428	430	432	434	434	436	437	439
	A (a.u.)	0.263	0.278	0.289	0.310	0.319	0.336	0.360	0.364
pH 7.0	λ_max_ (nm)	407	407	407	407	407	408	408	408
	A (a.u.)	1.495	1.475	1.478	1.495	1.474	1.497	1.485	1.490
pH 8.0	λ_max_ (nm)	403	404	404	404	405	405	405	405
	A (a.u.)	1.854	1.884	1.863	1.861	1.847	1.853	1.852	1.847
pH 9.0	λ_max_ (nm)	401	403	403	403	403	404	404	404
	A (a.u.)	2.052	2.075	2.071	2.052	2.062	2.046	2.051	2.040
pH 10.0	λ_max_ (nm)	402	403	403	403	404	404	404	404
	A (a.u.)	2.272	2.282	2.305	2.292	2.271	2.289	2.320	2.293
pH 11.0	λ_max_ (nm)	400	400	400	399	400	400	399	400
	A (a.u.)	2.804	2.789	2.794	2.785	2.780	2.802	2.789	2.822

**Table 2:** Comparison of data on the stability study of colloids reduced by malic acid determined using a UV-Vis spectrometer.

		Synthesis Day	1 Week	2 Weeks	3 Weeks	4 Weeks	5 Weeks	6 Weeks	7 Weeks
pH 7.0	λ_max_ (nm)	393	394	394	394	394	395	395	395
	A (a.u.)	0.830	0.870	0.875	0.873	0.880	0.879	0.891	0.902
pH 8.0	λ_max_ (nm)	397	397	397	397	397	398	398	398
	A (a.u.)	1.503	1.598	1.634	1.659	1.693	1.689	1.737	1.709
pH 9.0	λ_max_ (nm)	409	409	409	409	409	409	408	409
	A (a.u.)	2.661	2.601	2.626	2.661	2.662	2.706	2.697	2.657
pH 10.0	λ_max_ (nm)	422	422	422	422	422	422	422	422
	A (a.u.)	1.624	1.553	1.572	1.573	1.566	1.585	1.574	1.571
pH 11.0	λ_max_ (nm)	429	---	---	---	---	---	---	---
	A (a.u.)	0.112	---	---	---	---	---	---	---

## Supplementary Materials

The following are available online at https://www.mdpi.com/1996-1944/13/23/5444/s1. Figure S1: (Figure 2a). TEM image of the silver nanoparticles reduced by citric acid at pH 6.0, Figure S2: (Figure 2b). TEM image of the silver nanoparticles reduced by citric acid at pH 7.0, Figure S3: (Figure 2c). TEM image of the silver nanoparticles reduced by citric acid at pH 8.0, Figure S4: (Figure 2d). TEM image of the silver nanoparticles reduced by citric acid at pH 9.0, Figure S5: (Figure 2e). TEM image of the silver nanoparticles reduced by citric acid at pH 10.0, Figure S6: (Figure 2f). TEM image of the silver nanoparticles reduced by citric acid at pH 11.0, Figure S7: (Figure 7a). TEM image of the silver nanoparticles reduced by malic acid at pH 7.0, Figure S8: (Figure 7b). TEM image of the silver nanoparticles reduced by malic acid at pH 8.0, Figure S9: (Figure 7c). TEM image of the silver nanoparticles reduced by malic acid at pH 9.0, Figure S10: (Figure 7d). TEM image of the silver nanoparticles reduced by malic acid at pH 10.0, Figure S11: (Figure 7e). TEM image of the silver nanoparticles reduced by malic acid at pH 7.0, Figure S12: Distribution diagrams of the protonation of citric acid, Figure S13: Distribution diagrams of the protonation of malic acid.
