# Supplementary material for: The Effect of pH on the Size of Silver Nanoparticles Obtained in the Reduction Reaction with Citric and Malic Acids
Source: Materials (Basel). 2020 Nov 29;13(23):5444. doi: 10.3390/ma13235444 (PMC7730334; doi:10.3390/ma13235444)
Supplement: Supplementary file 1 [file materials-13-05444-s001.pdf]

materials

Supplementary Materials

The Effect of pH on the Size of Silver Nanoparticles Obtained in the Reduction Reaction with Citric and Malic Acids

Lukasz Marciniak [1], Martyna Nowak [1], Anna Trojanowska [2], Bartosz Tylkowski [2] and Renata Jastrzab [1,*]

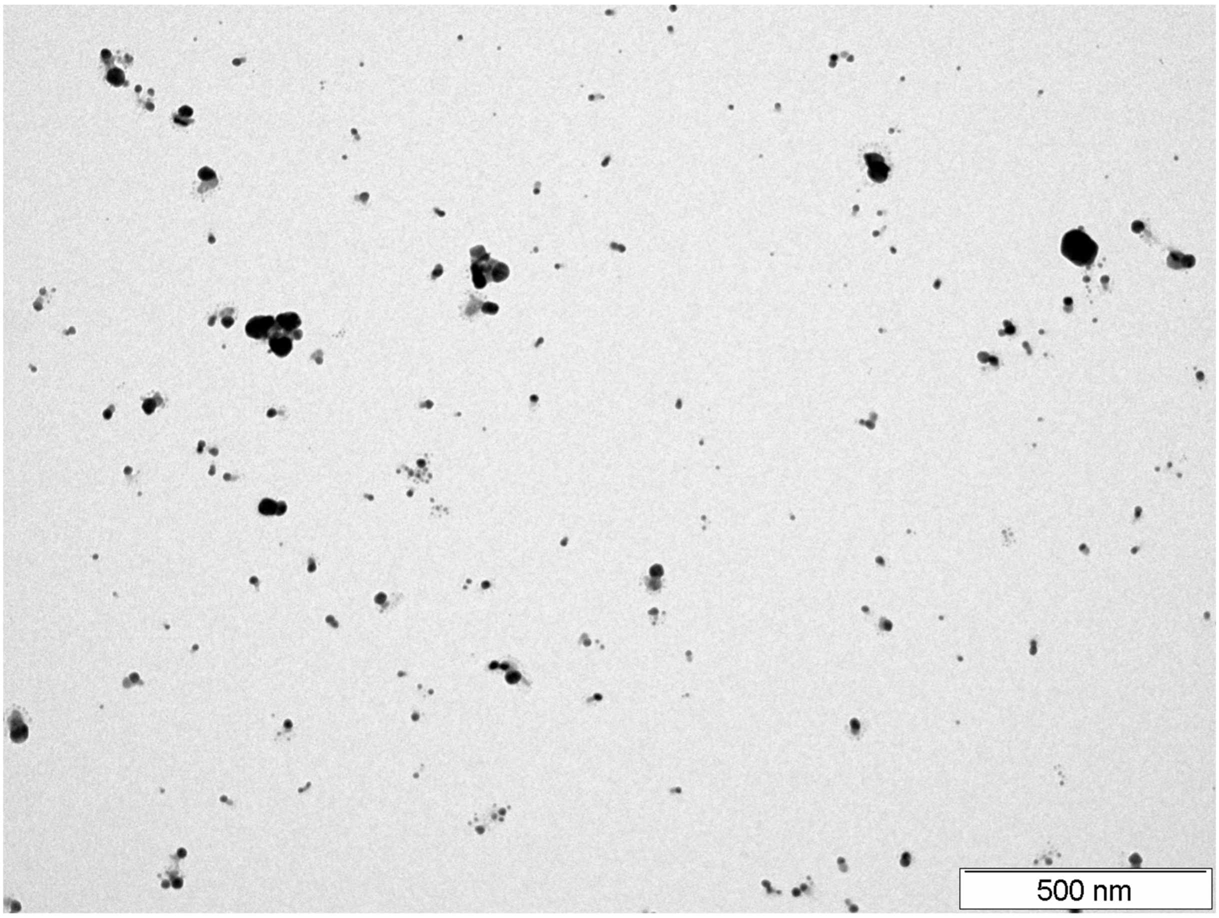

Figure S1. (Figure 2a). TEM image of the silver nanoparticles reduced by citric acid at pH 6.0.

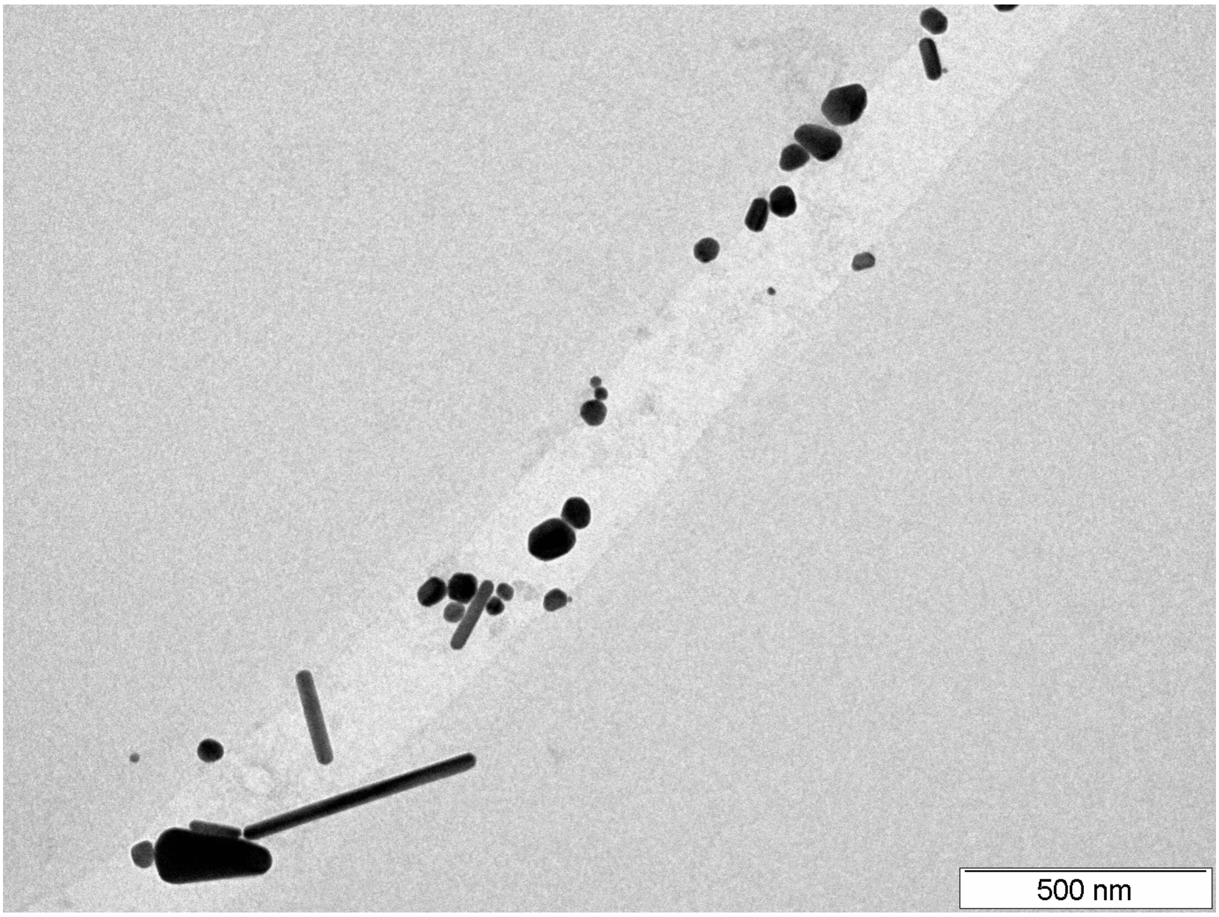

Figure S2. (Figure 2b). TEM image of the silver nanoparticles reduced by citric acid at pH 7.0.

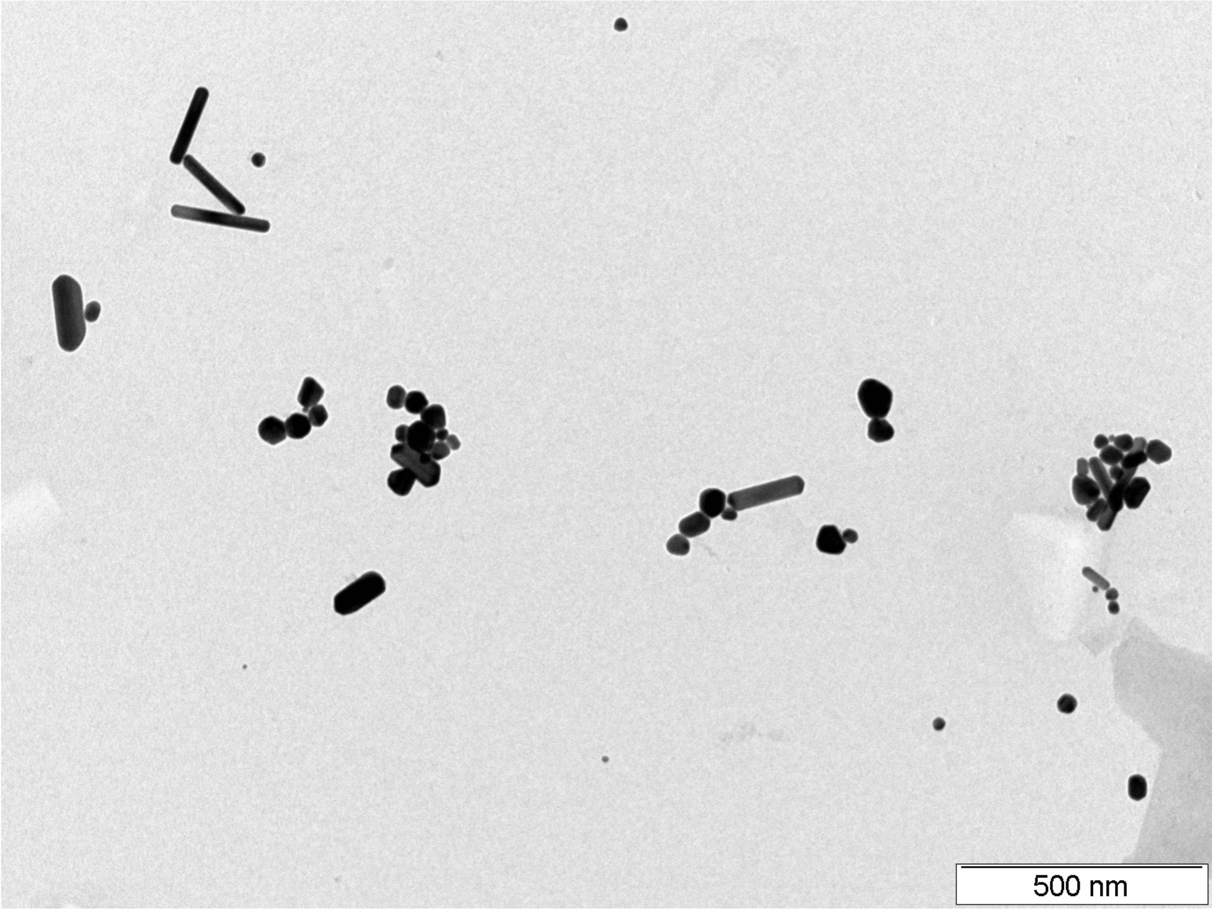

Figure S3. (Figure 2c). TEM image of the silver nanoparticles reduced by citric acid at pH 8.0.

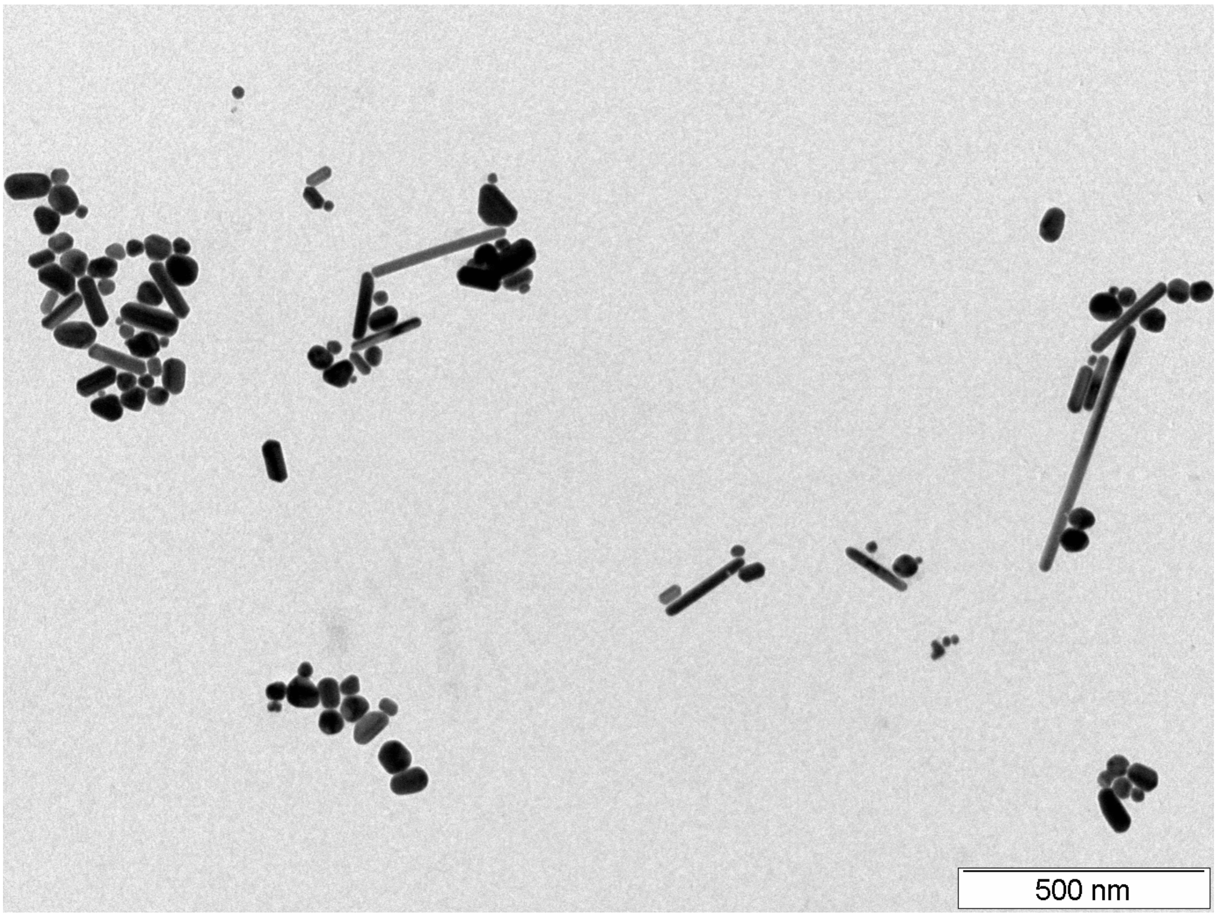

Figure S4. (Figure 2d). TEM image of the silver nanoparticles reduced by citric acid at pH 9.0.

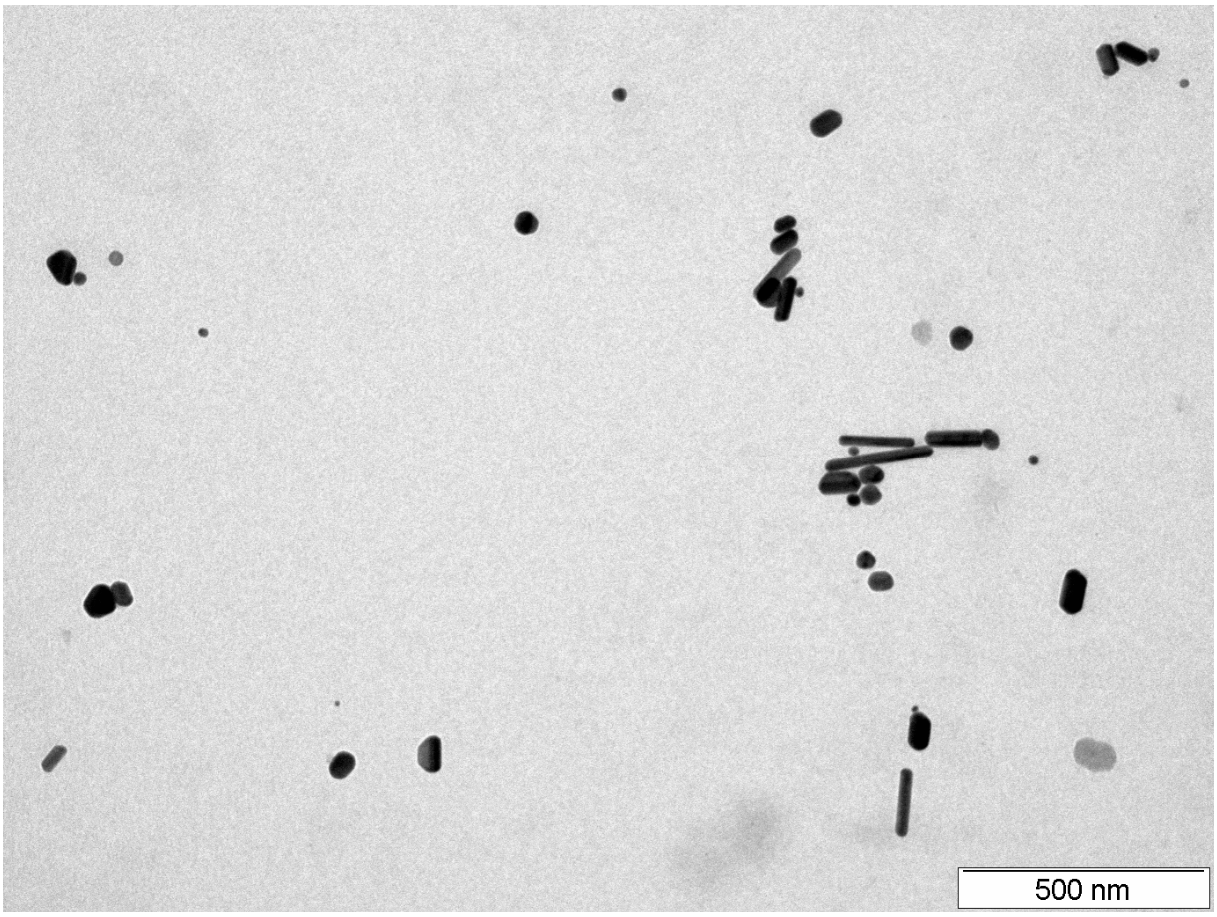

Figure S5. (Figure 2e). TEM image of the silver nanoparticles reduced by citric acid at pH 10.0.

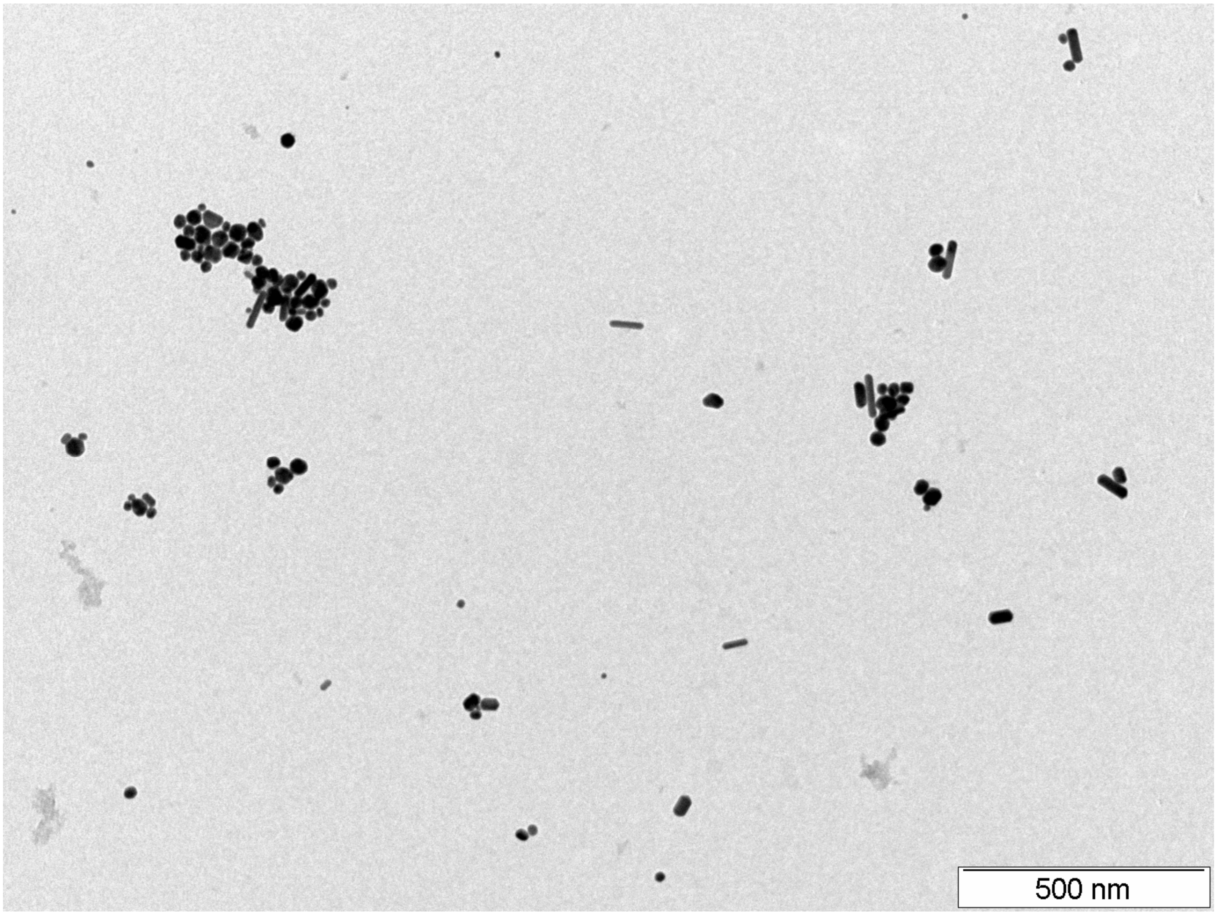

Figure S6. (Figure 2f). TEM image of the silver nanoparticles reduced by citric acid at pH 11.0.

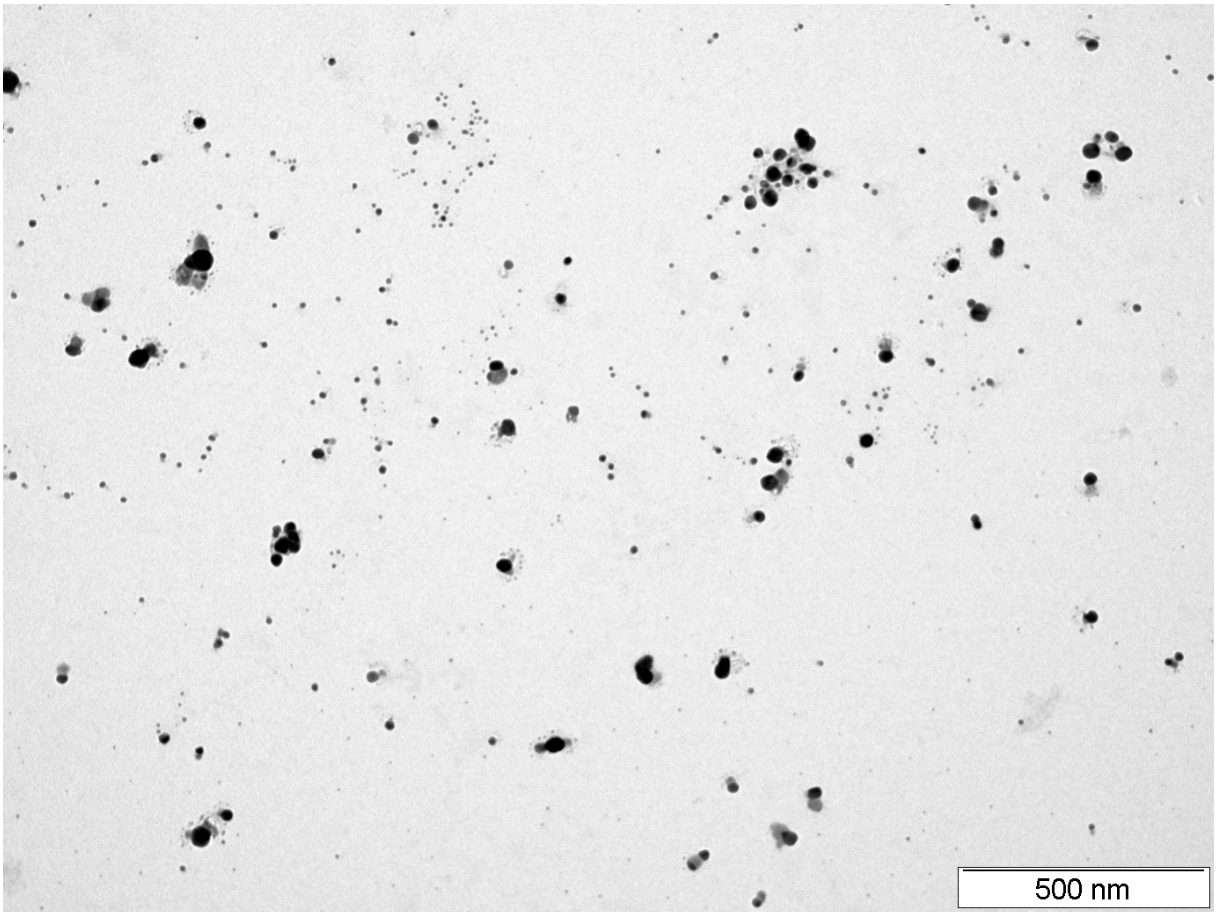

Figure S7. (Figure 7a). TEM image of the silver nanoparticles reduced by malic acid at pH 7.0.

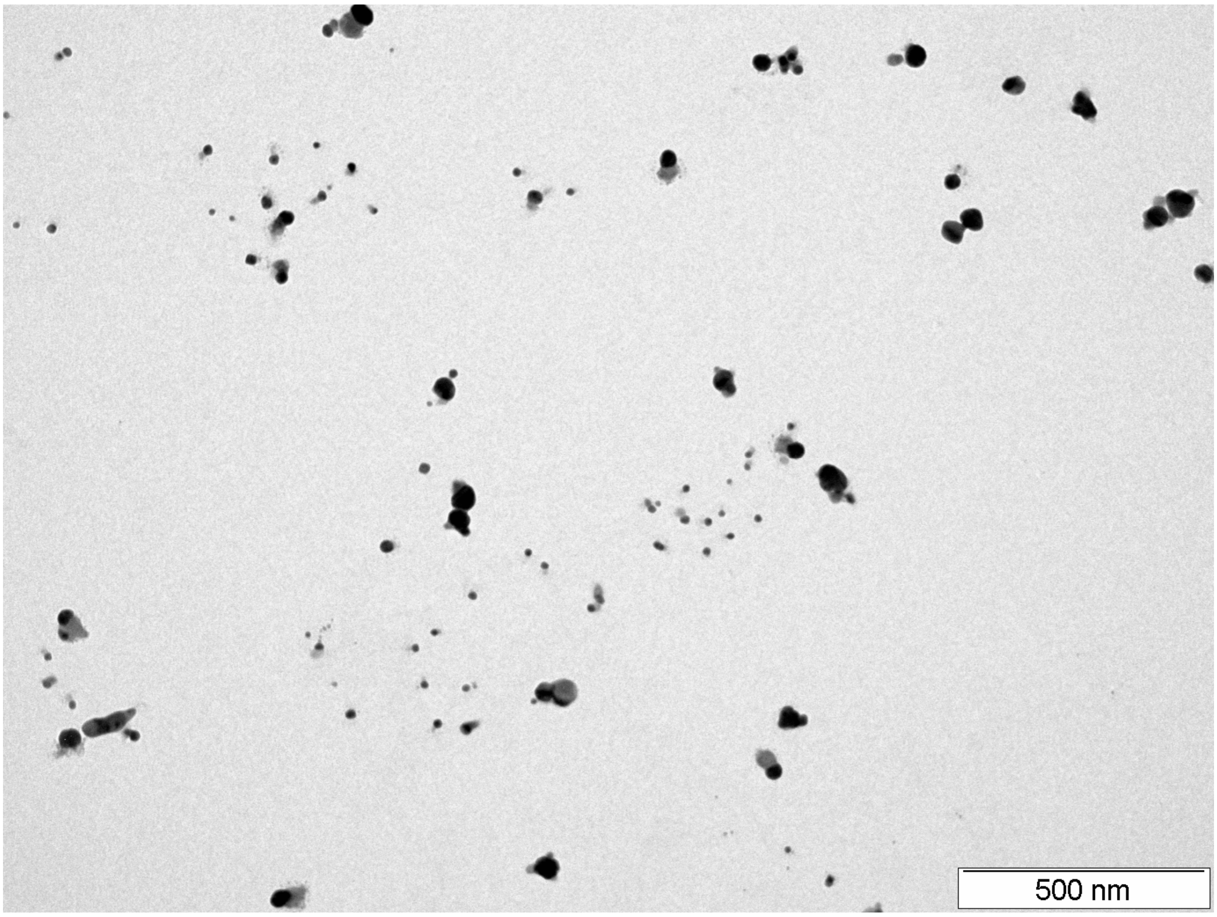

Figure 8S. (Figure 7b). TEM image of the silver nanoparticles reduced by malic acid at pH 8.0.

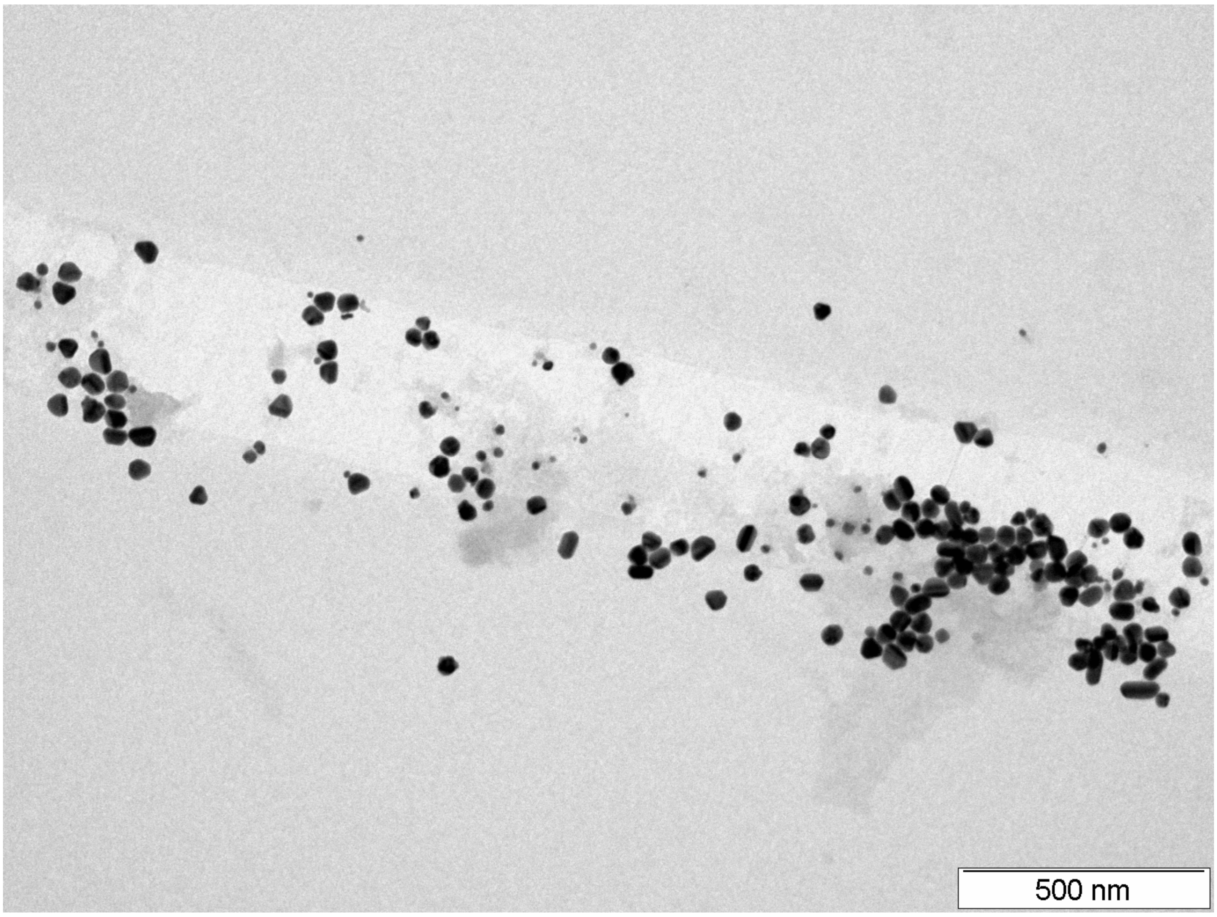

Figure S9. (Figure 7c). TEM image of the silver nanoparticles reduced by malic acid at pH 9.0.

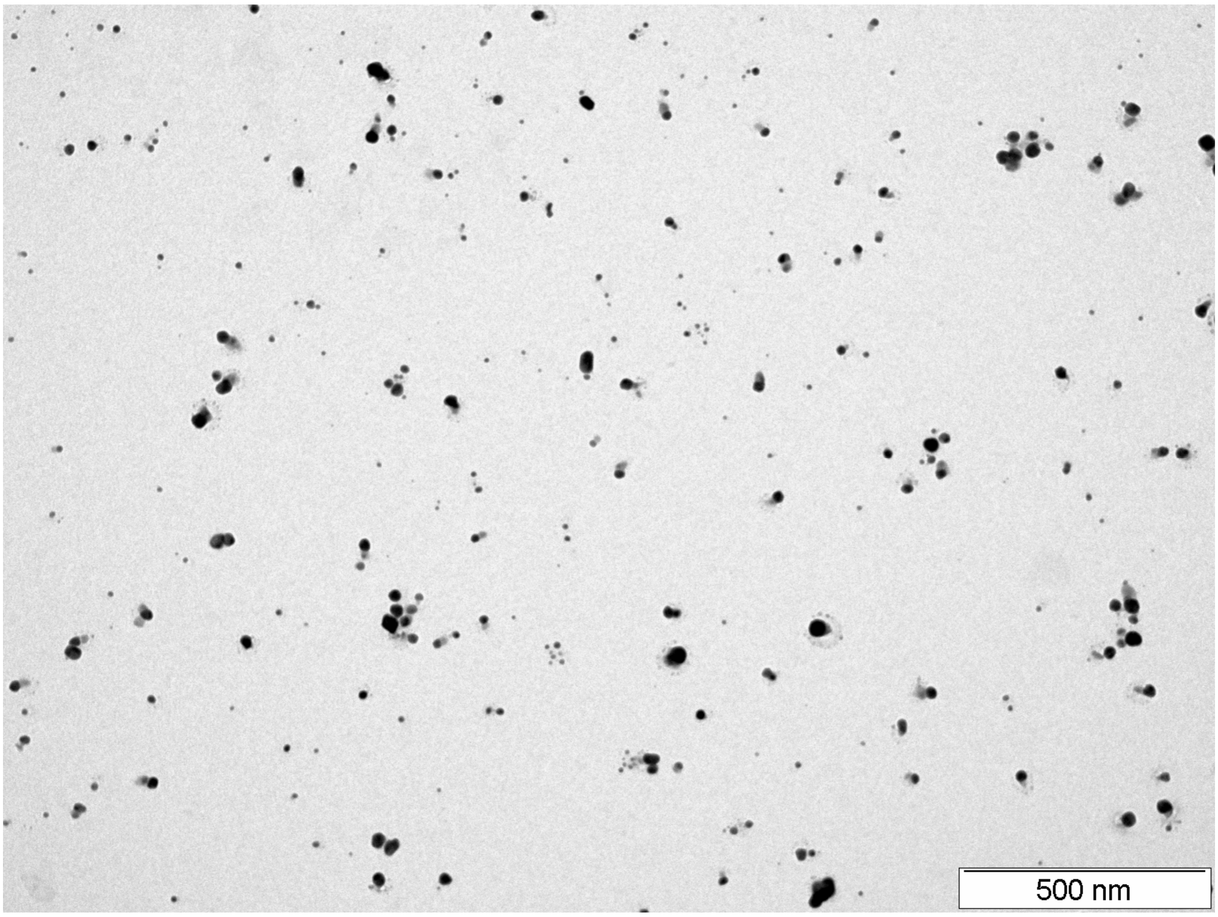

Figure S10. (Figure 7d). TEM image of the silver nanoparticles reduced by malic acid at pH 10.0.

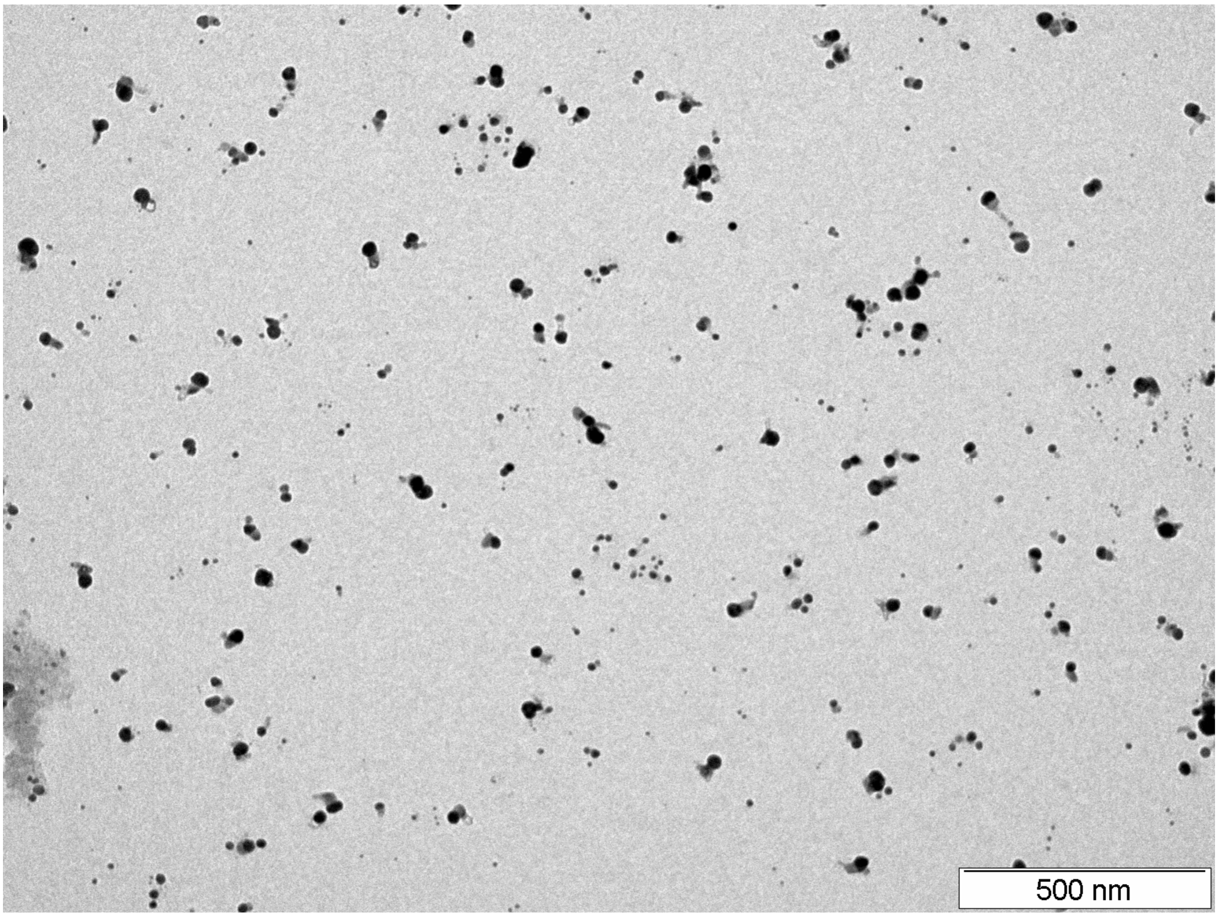

Figure S11. (Figure 7e). TEM image of the silver nanoparticles reduced by malic acid at pH 7.0.

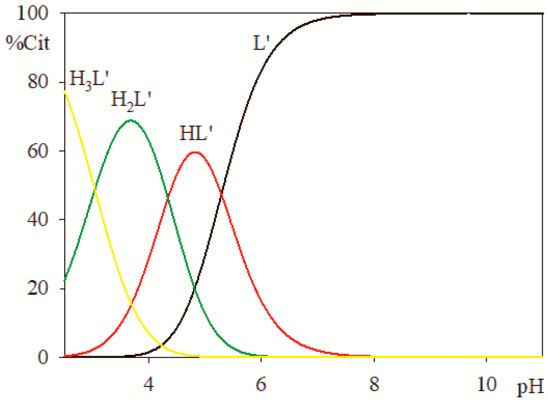

Figure S12. Distribution diagrams of the protonation of citric acid.

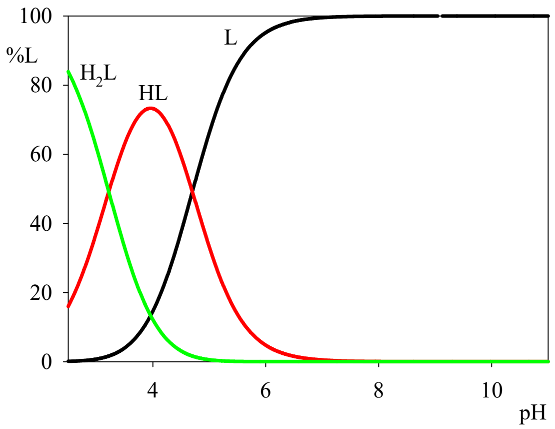

Figure S13. Distribution diagrams of the protonation of malic acid.

Publisher's Note: MDPI stays neutral with regard to jurisdictional claims in published maps and institutional affiliations.

© 2020 by the authors. Submitted for possible open access publication under the terms and conditions of the Creative Commons Attribution (CC BY) license (http://creativecommons.org/licenses/by/4.0/).
